# Aryl Hydrocarbon Receptor as an Anticancer Target: An Overview of Ten Years Odyssey

**DOI:** 10.3390/molecules28103978

**Published:** 2023-05-09

**Authors:** Hamza Hanieh, Mohammad Bani Ismail, Manal A. Alfwuaires, Hairul-Islam M. Ibrahim, Mahdi Farhan

**Affiliations:** 1 Basic Medical Sciences Department, Faculty of Medicine, Aqaba Medical Sciences University, Aqaba 77110, Jordan; 2 International Medical Research Center (iMReC), Aqaba 77110, Jordan; 3 Department of Biological Sciences, College of Science, King Faisal University, Hofuf 31982, Saudi Arabia; 4 Department of Drug Development, UniTechPharma, 1700 Fribourg, Switzerland

**Keywords:** aryl hydrocarbon receptor, ligands, immunotherapy, cancer

## Abstract

Aryl hydrocarbon receptor (AhR), a ligand-activated transcription factor belonging to the basic helix–loop–helix (bHLH)/per-Arnt-sim (PAS) superfamily, is traditionally known to mediate xenobiotic metabolism. It is activated by structurally diverse agonistic ligands and regulates complicated transcriptional processes through its canonical and non-canonical pathways in normal and malignant cells. Different classes of AhR ligands have been evaluated as anticancer agents in different cancer cells and exhibit efficiency, which has thrust AhR into the limelight as a promising molecular target. There is strong evidence demonstrating the anticancer potential of exogenous AhR agonists including synthetic, pharmaceutical, and natural compounds. In contrast, several reports have indicated inhibition of AhR activity by antagonistic ligands as a potential therapeutic strategy. Interestingly, similar AhR ligands exert variable anticancer or cancer-promoting potential in a cell- and tissue-specific mode of action. Recently, ligand-mediated modulation of AhR signaling pathways and the associated tumor microenvironment is emerging as a potential approach for developing cancer immunotherapeutic drugs. This article reviews advances of AhR in cancer research covering publication from 2012 to early 2023. It summarizes the therapeutic potential of various AhR ligands with an emphasis on exogenous ligands. It also sheds light on recent immunotherapeutic strategies involving AhR.

## 1. Introduction

Over the past decade, advances in understanding molecular oncology coupled with an improved molecular simulation have created a paradigm shift in anticancer drug discovery. These advances have provided a huge ray of new compounds approved as anticancer drugs [1,2], entering clinical trials [3,4], or being studied at the preclinical level [5,6]. When compared with traditional chemotherapies and anticancer drugs, contemporary anticancer compounds selectively interact with cellular proteins implicated in the malignant phenotypes. These compounds open a horizon for targeted anticancer therapies with reduced toxicity to meet the overwhelming needs.

A plethora of in silico and in vitro studies have identified myriad of synthetic and natural lead compounds interacting with specific proteins in the cell to exert anticancer effects. For example, derivatives of 1,3-thiazole [7], pyridine [8], and benzimidazole [9] inhibit the progression of multiple cancer cells by blocking the vascular endothelial growth factor receptor-2 (VEGFR-2). The pictilisib [10], gallic acid [11], samotolisib [12], and TAS-117 [13] exert anticancer potential by targeting one or more components of the phosphatidylinositol 3-kinase (PI3K)/protein kinase B (AKT)/mammalian target of rapamycin (mTOR) signaling pathway. In the context of targeting specific proteins in cancer cells by exogenous compounds, aryl hydrocarbon receptor (AhR), a ligand-activated transcription factor, exemplifies a unique pleiotropic target.

The AhR is activated by a broad range of synthetic, natural, and endogenous agonistic molecules to induce the expression of downstream genes involved in various physiological processes. For decades, AhR has been studied by toxicologists to unravel its roles in mediating environmental stimuli and toxicity of xenobiotic compounds such as 2,3,7,8-tetrachlorodibenzo-p-dioxin (TCDD) [14,15]. Recently, AhR has attracted the attention of immunologists and oncologists owing to its potential to modify numerous physiological processes through interaction with structurally diverse ligands [16,17,18,19]. The AhR is now experiencing a new lease of life as a molecular target for new therapeutic options in immune-related diseases and malignancies. For example, activation of AhR by certain agonistic compounds alters T cell differentiation [20,21], inflammation [22,23], autoimmune responses [24,25], and inflammation-associated tumorigenesis [26,27]. 

Accumulating evidence has suggested modulation of AhR signaling by agonistic or antagonistic compounds as a valuable therapeutic strategy to control cancer development and progression. It has been demonstrated that the AhR lead agonistic acrylonitrile (Z)-2-(3,4-dichlorophenyl)-3-(1H-pyrrol-2-yl)prop-2-enenitrile (ANI-7) and analogs inhibit proliferation of a broad panel of breast [28], ovarian and lung [29] cancer cells. It induces DNA damage, and checkpoint activation and makes the arrest of the cell cycle at the S-phase [28]. Furthermore, activation of AhR by indole-3-carbinol (I3C) suppresses tumorigenesis in the colon via regulating transcription of ring finger protein 43 (Rnf43), zinc, and ring finger 3 (Znrf3) and E3 ubiquitin ligase [30].

Interestingly, anticancer effects of ligand-mediated inhibition of AhR activation have been reported. For instance, the AhR antagonist *N*,2-dimethyl-*N*-[1,2-dimethylindol-5-yl]quinazoline-4-amine (compound **12**) inhibits proliferation in estrogen receptor (ER)-positive breast cancer cells by reducing the levels of cyclin-dependent protein kinase (CDC2) and cell division cycle 25c (CDC25c) and increasing that of cyclin B1 [31]. Inhibition of AhR signaling by 2-((2-(5-bromofuran-2-yl)-4-oxo-4*H*-chromen-3-yl)oxy)acetamide suppresses migration and invasion of TNBCs (triple negative breast cancer cells) [32]. Remarkably, modulation of AhR activity by small molecules alters vital signaling pathways associated with cancer development and progression such as Wnt/β-catenin [30], PI3K/AKT [33], and mitogen-activated protein kinase (MAPK)/extracellular signal-regulated kinase (ERK) [34] signaling pathways.

A wide range of structurally diverse AhR ligands has been evaluated for therapeutic potential in various cancers. This progress has created a multiple cell- and ligand-specific modulation of cancer progression which sparks an interest in AhR as a therapeutic target. In this article, we review the recent advances of AhR research covering articles published from 2012 to early 2023. We summarize the structure and pathways of AhR, its roles in cancer, and the ligand-mediated modulation of AhR functions in different cancers with special emphasis on synthetic, pharmaceutical, and natural Ahr ligands. Recently, modulation of AhR signaling has attracted interest in the development of cancer immunotherapies [35,36,37]. Thus, in this review, we shed light on the modulation of AhR signaling in cancer immunotherapy as a potential therapeutic strategy. 

## 2. AhR Structure and Activation

**Figure 1:**
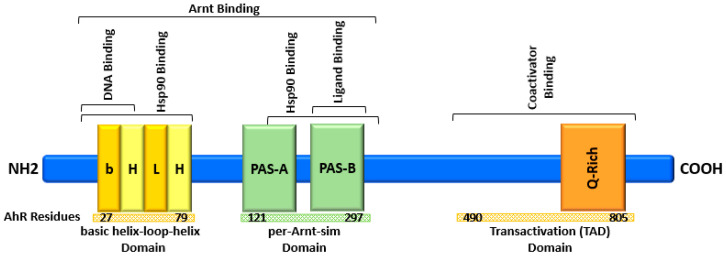
Schematic illustration of AhR domains and binding sites.

The AhR is a conserved transcription factor from invertebrates and is broadly expressed in body tissues [38,39,40]. It belongs to the basic helix-loop-helix (bHLH)/per-Arnt-sim (PAS) superfamily; it is the only known member to be ligand-activated. The N-terminal of AhR contains the bHLH motif, which has two functionally different and highly conserved regions (Figure 1). The basic “b” region is where the AhR binds to DNA, and the second is the helix “HLH” which promotes dimerization with other proteins. The AhR embraces two repeated PAS domains, the PAS-A and PAS-B. The PAS-A is involved in AhR heterodimerization with other proteins, and PAS-B mediates AhR interaction with ligands. Notably, PAS-B contains numerous conserved residues that are essential for interaction with diverse compounds [41,42]. Furthermore, PAS-B is implicated in the interaction of AhR with heat shock protein 90 (Hsp90) to determine the binding affinity and specificity of the ligands [43]. The C-terminal of AhR contains TAD (transactivation domain), also known as the Q-rich domain (Figure 1), that is responsible for interaction with cofactors and mediating transcriptional activation [44]. For decades, understanding the ligand binding of AhR has been hindered by the scarcity of three-dimensional structures of the PAS-B domain. Therefore, intensive research efforts have been directed toward developing crystal structures for AhR. Recently, Dai and colleagues have presented several crystal structures of drosophila PAS-B domain bound to α-naphthoflavone (ANF) and characterized the binding pocket [45]. They have also presented crystal structures of mouse AhR nuclear translocator (Arnt) bound to the drosophila PAS-B domain [45]. Importantly, the crystal structure of the human AhR has been recently developed [46]. This structure reveals a unique organization of the ligand-binding pocket in the PAS-B domain and introduces the structural elements of the binding specificity. Furthermore, they have presented a structure of a complex including chaperone Hsp90 and the co-chaperone XAP2 (AhR-associated protein 9; ARA9) and indirubin-bound AhR [46]. Collectively, these significant advances in developing three-dimensional structures provide the basis for future identification of specific AhR ligands, illustration of mechanistic details, and consequently, drug design for targeted therapies. 

**Figure 2:**
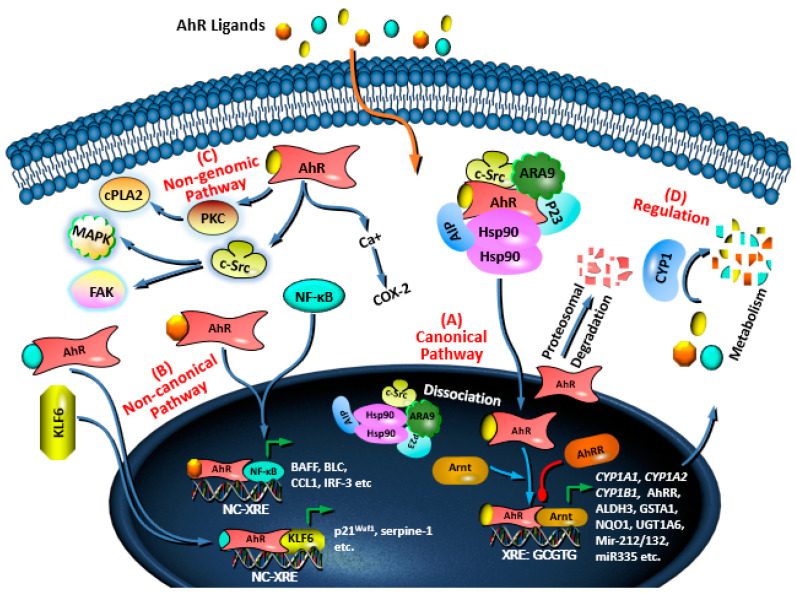
AhR signaling pathways and regulatory mechanisms. (**A**) The canonical AhR pathway showing binding of AhR/Arnt complex to the XRE sequence; (**B**) The non-canonical AhR pathway showing binding other AhR complexes to non-XRE sequences; (**C**) The non-genomic AhR pathway showing activation of certain cellular proteins; (**D**) Regulation of AhR activation by proteasomal degradation of AhR and metabolism of the ligands.

Upon binding to an agonistic compound in the cytoplasm, conformational changes occur in AhR leading to exposure of the nuclear localization sequence (NLS) [47]. Subsequently, AhR interacts with importin-β to translocate into the nucleus where it dimerizes with other proteins, typically Arnt [39,48]. As illustrated in Figure 2, forming this heterodimer dissociates AhR from Hsp90, c-Src kinase, and the co-chaperones p23, ARA9, and AhR interacting protein (AIP) in the nucleus [49]. This series of events transforms AhR into its transcriptionally active form and endows it with DNA binding capacity. The AhR after that binds to a specific DNA penta-nucleotide sequence (3′-GCGTG-5′), called xenobiotic responsive element (XRE), and variable flanking nucleotides that include 5′-CATG{N6}C[T|A]TG-3′ [50]. This interaction leads to transcriptional activation of the downstream genes [42,51].

## 3. AhR Signaling Pathways and Regulation

The AhR controls the expression of a battery of downstream genes through its canonical and non-canonical pathways. Through the canonical pathway (Figure 2A), the AhR/Arnt complex binds to the XRE sequence upstream of genes such as AhR repressor (*AhRR*) and xenobiotics metabolizing enzymes including cytochrome P450 1A1 (*CYP1A1*), *CYP1A2*, *CYP1B1* [52,53]. The canonical AhR downstream genes also include phase II enzymes such as glutathione-S-transferase A1 (*GSTA1*), aldehyde dehydrogenase (*ALDH3*), quinone oxidoreductase 1 (*NQO1*), uridine 5′-diphosphate-glucuronosyltransferase 1A6 (*UGT1A6*) [54,55], and interleukin-22 (*Il22*) [56]. More recently, microRNAs (miR)-encoding genes have been added to the list of canonical AhR downstream including *miR-132* and *miR-212* [57,58], *miR-335* [59], *miR-543-3p* [60], and *miR-150-5p* [60,61]. 

In the presence of certain agonistic compounds, AhR forms heterodimers with proteins other than Arnt. This leads to the expression of downstream genes through non-XRE sequences (Figure 2B). For example, AhR forms a complex with nuclear factor-κB (NF-κB) and induces the expression of several immune response-related proteins such as B cell-activating factor (BAFF), B lymphocyte chemoattractant (BLC), transcription factor interferon responsive factor-3 (IRF-3) and CC-chemokine ligand 1 (CCL1) [53,62]. In addition, AhR directly interacts with KLF transcription factor 6 (KLF6), and regulates the expression of protein p21^Waf1^ and serpine-1 through binding to a non-XRE sequence [63,64].

Activation of AhR by agonists induces non-genomic pathways leading to the modulation of different physiological processes (Figure 2C). Activated AhR increases the concentration of intracellular calcium and regulates the kinase activities of focal adhesion kinase (FAK)/c-Src and MAPK [65,66], and protein kinase C (PKC) [66]. The increase in the intracellular calcium ions promotes activation of cytosolic phospholipase A2 (cPLA2) and cyclooxygenase 2 (COX2) [50,67]. Furthermore, AhR activates c-Src, and the c-Src then contributes to the phosphorylation of epidermal growth factor receptor (EGFR) and indoleamine-2,3-dioxygenase 1 (IDO1) [68].

The AhR signaling pathways are tightly regulated by different mechanisms in the cytoplasm and the nucleus (Figure 2D). These mechanisms potentiate, inhibit, or drive signaling to the genomic or non-genomic pathways. The first mechanism occurs in the cytoplasm where chaperons and co-chaperons form a complex with AhR to keep it inactive [14]. Other mechanisms in the cytoplasm include the E3 ubiquitin-mediated proteasomal degradation of AhR and degradation of AhR ligands by phase I xenobiotic metabolizing enzymes. In the nucleus, the prolonged transcriptional activity of the AhR–Arnt complex is disrupted by AhRR that competes with AhR to dimerize with Arnt and stops the transcriptional activity [42].

## 4. Role of AhR in Cancer at Glance

A multitude of epidemiological and experimental studies has expanded the physiological roles of AhR from being a mediator of environmental stimuli to a significant player in malignancy. Several models of AhR inhibition or overexpression have demonstrated that the constitutive AhR displays varying activities in cancer ranging from tumor-promoting to tumor-suppressing activities. It has been shown that inhibition of AhR by RNA interference in breast cancer cells either boosts the proliferation of BT474 (ER-positive) or had no effects in MDA-MB-468 (ER-negative) cells [69]. Deletion of AhR enhances the invasive capacity of the MDA-MB-231 breast cancer cells, but on the contrary decreases cell proliferation and proliferation-related genes [70]. Furthermore, deletion of AhR in the colon cancer cells HCT116 and HT29 represses proliferation in a cell-specific manner [71]. This was concomitant with altered cell cycle progression, decreased ATP production, suppression of fatty acids biosynthetic pathway, and reduced expression and/or activity of the components of the PI3K/AKT pathway [71]. The AhR either suppresses clonogenic potential or augments differentiation of cancer stem cells (CSCs) to exert tumor suppressor activities. It suppresses the expression of the pluripotency factors such as ALDH1, BMI1 proto-oncogene (BMI1), CD133, and MUSASHI-1 and increases the expression of differentiation factors [72,73,74]. Finally, higher expression of AhR in malignancies such as hepatic [75] and lung [76] cancers is likely associated with poor outcomes. Collectively, these and other observations clearly indicate that the function of constitutive AhR varies and is cancer- and cell-specific. Furthermore, it is plausible to conclude that certain levels of AhR signaling are necessary for normal physiological functions, while increased or inadequate signaling may promote malignancies. Although several studies have indicated the tumor-enhancing role of constitutive AhR, more cancer-specific studies are required. Yet, AhR offers a valuable opportunity to develop cancer-specific and/or cell-specific AhR-dependent therapies. 

The AhR regulates complicated transcriptional processes through interactions with cancer-associated signal transduction pathways. These pathways include transforming growth factor-β (TGF-β), PI3K/AKT/mTOR, NF-кB, FAK/c-Src, and Wnt5a/b-β-catenin, reviewed in [42]. It has been demonstrated recently that AhR inhibits TGF/SMA-and MAD-related protein 3 (SMAD3) signaling in medulloblastoma mouse models to prevent cell proliferation and differentiation [77]. Moreover, the ablation of AhR in TNBCs correlates with the expression of Wnt5a/b and β-catenin signaling molecules [78]. Notably, the interaction of AhR with cancer-associated signaling pathways has been studied also by using its ligands. For instance, ligand-activated AhR inhibits PI3K/AKT signaling pathways accompanied by reduced cyclin D1/D3 and cell division protein kinase 4 (CDK4) in breast cancer cells [79]. The activated AhR also induces the MAPK/ERK pathway concomitant with elevated levels of p21^Waf1^ in an ERα-dependent manner [79]. 

In addition, ligand-induced AhR signals in lung cancer cells upregulate the expression of osteopontin (OPN) through AhR and NF-κB pathways interplay, and inhibition of AhR by the antagonist desmosflavone (DMF) reverses these effects [80]. In hepatocarcinoma, activated AhR regulates long interspersed nuclear element-1 (Line-1) expression via the canonical TGF-β1 signaling pathway and associated epigenetic mechanisms [81]. Zhu and coworkers have depicted that the partial AhR agonist 3,3′-diindolylmethane (DIM) modulates AhR signaling to inhibit Ras homolog family member A (RhoA)/Rho-associated protein kinase 1 (ROCK1) pathway, and consequently COX2/prostaglandin E2 (PGE2) pathway [82]. Moreover, inhibition of ER signaling by AhR ligands was previously reported [83]. This inhibition occurs via different mechanisms including direct suppression through anchoring AhR/Arnt heterodimer to inhibitory XRE (iXRE) in ER target genes, silencing of common coactivators such as Arnt, extended proteasomal degradation of ER, and altered estrogen metabolism [83]. The activated AhR interacts with Src and controls its activity [84]. They have also found that the Src-mediated crosstalk between AhR and EGFR leads to activation of ERK1/2, and consequently stimulating cell proliferation in colon cancer [84].

## 5. AhR Ligands in Cancer

**Figure 3:**
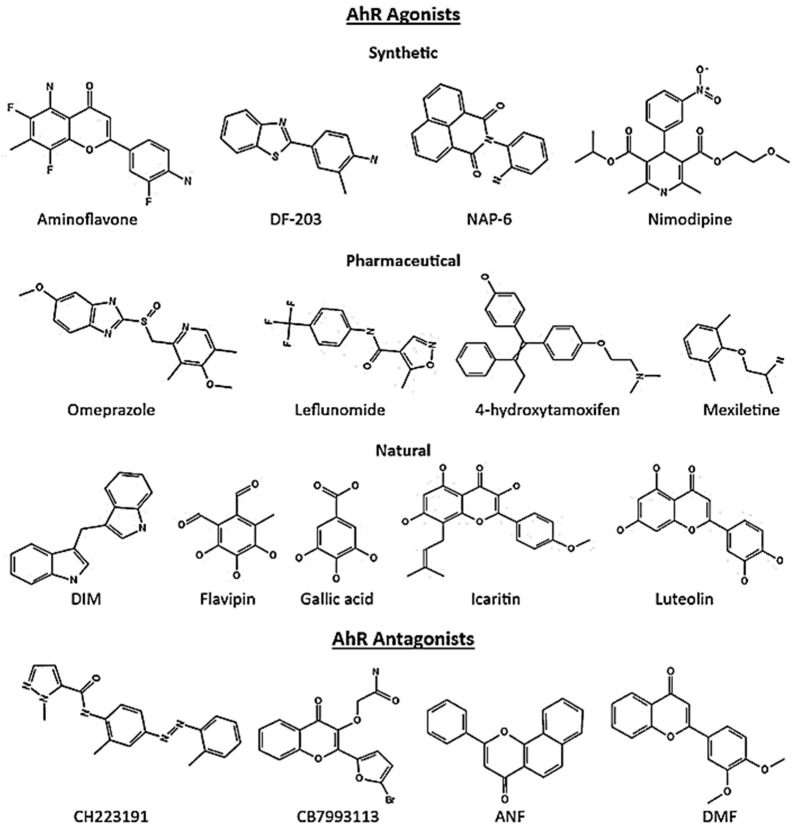
Representative structures of AhR exogenous ligands.

Initially, the xenobiotic compound TCDD, PAHs (polycyclic aromatic hydrocarbons), and HAHs (halogenated aryl hydrocarbons) were identified as the principal sources of exogenous AhR ligands. However, it was soon recognized that an array of compounds binds to the receptor, namely selective AhR modulators (SAhRMs), and influence AhR functions in a cell- and ligand-specific manner. Currently, it is evident that the sources of SAhRMs include structurally diverse synthetic, pharmaceutical, phytochemical, and endogenous compounds that act as agonists, antagonists, or partial agonist/antagonist [42,85]. Figure 3 presents representative structures of AhR ligands. The large binding site of AhR includes diverse interacting residues with hydrophobic contacts and many conformations [50,86,87], which may contribute to the promiscuity of AhR molecular recognition to ligands. These features of the AhR binding site and its residues make the interaction with ligands including SAhRMs primarily hydrophobic with van der Waals’ dimensions of 14 × 12 × 5 Å [88,89]. 

Currently, diverse classes of synthetic compounds have been identified as AhR ligands with anticancer properties such as aminoflavones, aminobenzothiazoles, aminoglycoside, naphthylamides and acrylonitriles. Some of these synthetic AhR ligands are approved as anticancer drugs [2], entering clinical trials [4,90], or being studied at the preclinical level [5,6]. A screening study examining 596 pharmaceuticals for their AhR activity introduced a number of SAhRMs capable of activating AhR signaling [91]. They identified nine different AhR agonists, of which six agonists are approved by the U.S. Food and Drug Administration (FDA) including omeprazole, nimodipine, leflunomide, atorvastatin, flutamide, and mexiletine [91]. Later studies indicated anticancer effects of these pharmaceutical SAhRMs in breast cancer cell lines [59,92]. This activation leads to cell killing by inducing downstream cellular damage and pathways. In addition, phytochemical-derived AhR ligands including flavonoids, polyphenolics, heteroaromatics exhibit promising therapeutic properties in various cancers [93]. 

Several AhR ligands, in particular the agonists, exert their anticancer effects by acting largely or in part via metabolic activation of CYP1A1 and other metabolizing enzymes. For example, certain AhR agonists belonging to aminobenzothiazoles, aminoflavones, and naphthylamide may act through metabolic activation that induces cytotoxic downstream pathways such as oxidative stress leading to cell killing [18]. Furthermore, certain AhR ligands such as the quinazoline derivative (compound **12**) inhibit the microtubule polymerization [31]. 

In fact, several in vitro and in vivo studies indicate pro- and anti-cancer effects of both AhR agonistic and antagonistic ligands in different tissues. Mechanistically, this dichotomy may be attributed to differences in the structure of AhR ligands, the interacting residues of PAS-B, and the cells/tissue used to evaluate the ligand. From the ligand perspective, variability in the structure and potency of AhR ligands leads to activating AhR differentially, and consequently selectivity of gene expression. It has been reported that TCDD and the related less potent AhR ligand 2,3,7,8-tetrachlorodibenzofuran (TCDF) [94,95], and 3,3′,4,4′,5-pentachlorobiphenyl [95] induce the expression of different gene clusters in hepatic tissue. Furthermore, while the AhR ligand resveratrol induces weak expression of CYP1A1 and activates the expression of paraoxonase 1 (PON1) in the human hepatocellular cell line, TCDD induces strong expression of CYP1A1 but does not activate PON1 expression [96]. Moreover, differences in the interaction of various ligands with different PAS-B residues may contribute to differential modulations of AhR functions. It has been demonstrated that specific residues of PAS-B including H285, F289, F318, and H320 contribute to ligand-specific modulation of AhR functions [97]. Furthermore, the potency of TCDD and selected PHAs and HAHs to activate the AhR is decreased by mutation of H285 [98]. Interestingly, dynamic simulations have identified flexible segment (residues 307–329) of human PAS-B that exhibits distinct conformations and may act as a switch between the agonistic and antagonistic activity of AhR ligands [99], and thus, inducing/inhibiting the expression of different gene clusters. Collectively, the variable structure of AhR ligands interacts with different PAS-B residues with variable potency. Such varying interactions contribute to gene expression selectivity that may contribute, in addition to the cell-specific response, to the reported dichotomy about pro- and anti-cancer activities of AhR ligands. 

### 5.1. Breast Cancer

Extensive research using a broad panel of breast cancer cells has clearly indicated modulation of AhR functions by AhR ligands, including SAhRMs, as a promising strategy for drug development. This research progress has introduced a plethora of novel compounds that exert anti-breast cancer effects in ER-positive and TNBCs (Table 1). For example, the naphthylamide (2-(2-aminophenyl)-H-benzo[d,e]isquinoline-1,3[2H]-dione (NAP-6) is a newly identified AhR agonist with anti-breast cancer properties [100]. It induces death in MDA-MB-468 cells by enhancing the expression of H2AXγ and checkpoint kinase 2 (CHK2) and inducing cell cycle arrest at S-phase [100]. Comparable cytotoxic activities of NAP-6 and the AhR agonist 10-chloro-7H-benzo[de]benzo[4,5]imidazo[2,1-a]isoquinolin-7-one (10-Cl-BBQ) have been reported in MDA-MB-468, T47D, ZR-75-1 and SKBR3 breast cancer cell lines [101]. In their study, they demonstrated that the naphthalene moiety and an ortho-substituent on the N-phenyl moiety in naphthylamide are important for the biological activity [101].

Moreover, the acrylonitrile (*Z*)-2(3,4-dichlorophenyl)-3-(1*H*-pyrrol-2-yl) (ANI-7) and analogues are AhR agonists that inhibit proliferation in broad panel of breast cancer cell lines including MDA-MB-231, MDA-MB-468, ZR-75-1, SKBR3, MCF-7, BT474, T47D, and BT20 [100,101]. Interestingly, Stanton and colleagues predicted variable cytotoxic effects of a series of 2-phenylacrylonitriles as AhR ligands in MCF-7 cells using a generated predictive model for cytotoxicity [102]. The aminoglycoside 9-chloro-2-(furan-2-yl)-[1,2,4]triazolo[1,5-c]quinazolin-5-amine (CGS-15943) activates AhR signaling and prompts apoptosis in MDA-MB-486 breast cancer cells through upregulation of Fas ligand (FasL) [103]. It has been also shown that treating MCF-7 cells with AhR agonists including 7,12-dimethylbenz[a]anthracene (DMBA), 3-Methylcholanthrene (3MC), and benzo[a]pyrene (Bap) strongly inhibit mammosphere formation of the stem cells via AhR [104]. Interestingly, other agonists used in the study exerted weaker inhibition on mammosphere formation, suggesting differential selectivity of AhR agonists [104]. We previously found that TCDD inhibits proliferation, migration, and invasion of MDA-MB-231 and T47D cells and inhibits metastasis and tumor growth in a mouse model [58]. Our data showed that TCDD-activated AhR directly induced the expression of SRY-box transcription factor 4 (SOX4)-targeting the miR-212/132 cluster [58]. In a comparable study, TCDD and the partial AhR agonist 1,3,8-trichloro-6-methyldibenzofuran (MCDF) exerted inhibitory potential in MDA-MB-231 and BT474 cells in vitro and in nude mice inoculated with MDA-MB-231 [59]. These ligands downregulated the expression of SOX4 by inducing the expression of miR-335 [59]. In addition, it was also found that TCDD induced the expression of circRNA-BARD1 (circ_0001098), which inhibited breast cancer tumorigenesis via miR-3942-3p/BARD1 axis [105]. This axis blocked the cell cycle, promoted cell apoptosis, and suppressed the growth and metastasis of tumors in vivo [105].

Known pharmaceuticals have been identified as AhR ligands with anti-breast cancer activities. For instance, the anti-estrogen raloxifene is an AhR agonist that induces apoptosis in MDA-MB-231 cells and increases survival in metastasis animal model [106]. In a study screened the anticancer activities of AhR pharmaceutical agonists including leflunomide, omeprazole, sulindac, nimodipine, 4-hydroxytamoxifen, mexiletine, flutamide, and tranilast, only omeprazole decreased metastasis of MDA-MB-231 cells in a mouse model, and decreased expression of matrix metalloproteinase-9 (MMP-9) and C-X-C chemokine receptor 4 (CXCR4) [92]. Interestingly, using the same nine pharmaceuticals, all, but not 4-hydroxytamoxifen and mexiletine, suppressed migration of MDA-MB-468 cells [107]. These and other findings provide further confirmation of the ligand structure- and cell-specific actions of AhR ligands.

Several natural AhR ligands with anti-breast cancer potential have been reported. In our hands, we identified the polyphenolic compounds 3,4,5-trihydroxy-6-methylphthalaldehyde (flavipin) and 3,4,5-trihydroxybenzoic acid (gallic acid) as new AhR agonists [108,109]. These agonists suppress proliferation, migration, and invasion of MDA-MB-231 and T47D cells, reduce the levels of B-cell lymphoma-2 (BCL-2), and induce the expression of SOX4-targeting miR-212/132 cluster [108,109]. In addition, gallic acid induces apoptosis, increases the p53 level, and reduces that of cyclooxygenase-2 (COX-2) [109]. Importantly, the reported effects of flavipin and gallic acid were partially reversed with the depletion of AhR by RNA interference [108,109].

**Table 1:** Exogenous AhR ligands in breast cancer.

Compound	Ligand	Response	Cells	Refs.
TCDD	Agonist	Proliferation, Migration, Invasion Metastasis	MDA-MB-231, T47D	[58]
MCDF	Agonist	Invasion	MDA-MB-231, BT474	[59]
NAP-6	Agonist	Proliferation, Cell cycle, Checkpoint, DNA damage	MDA-MB-468, MDA-MB-231, ZR-75-1, SKBR3, T47D, MCF-7, BT474, BT20	[100]
10-Cl-BBQ	Agonist	Proliferation	MDA-MB-468, T47D, ZR-75-1, SKBR3	[101]
CGS-15943	Antagonist	Apoptosis	MDA-MB-486	[103]
ANI-7	Agonist	Proliferation, DNA damage, Cell cycle, Checkpoint	MDA-MB-468, MDA-MB-231, ZR-75-1, SKBR3, T47D, MCF-7, BT474 and BT20	[28,101]
13f (acrylonitrile)	Agonist	Proliferation	MCF-7	[29]
Compound **12** (quinazoline)	Antagonist	Apoptosis, Cell cycle arrest, Growth	MCF-7	[31]
2-phenylacrylonitriles (analogues)	Agonist	Proliferation (Predicted)	MCF-7	[102]
FDI-6	Agonist	Tumorsphere formation	MCF-7	[110]
CB7993113	Antagonist	Migration, Invasion	BP1, Hs578T, and SUM149	[32]
CH223191	Antagonist	Growth, Migration	MDA-MB-231	[111]
DMBA	Agonist	Migration, Invasion	BP1, Hs5787	[32]
Bap, 3MC,	Agonist	Mammosphere	MCF-7	[104]
5F-203	Agonist	DNA damage, Single strand breaks (SSBs)	MD-AMB-468	[112]
Raloxifene	Agonist	Apoptosis	MDA-MB-231	[103]
Omeprazole	Agonist	Invasion, Metastasis	MDA-MB-231	[92]
Leflunomide	Agonist	Migration	MDA-MB-468	[107]
Sulindac	Agonist	Migration	MDA-MB-468	[107]
Nimodipine	Agonist	Migration	MDA-MB-468	[107]
Flutamide	Agonist	Migration, Proliferation	MDA-MB-468, MCF-7	[107,113]
Tranilast	Agonist	Migration	MDA-MB-468	[107]
Flavipin	Agonist	Proliferation, Migration, Invasion	MDA-MB-231, T47D	[108]
Gallic acid	Agonist	Proliferation, Migration, Invasion, Growth	MDA-MB-231, T47D	[109]
Luteolin	Agonist	Migration, Growth, Metastasis	MDA-MB-231	[114]
Icaritin	Agonist	Growth	MCF-7	[115]
DIM	Agonist	Proliferation, Migration, Invasion, Growth	MDA-MB-231, T47D	[58,116]
Galangin	Antagonist	Proliferation, Apoptosis	MCF-7	[117]

Flavonoids such as 3′,4′,5,7-letrahydroxyflavone (luteolin) reduce the viability and induces apoptosis in MDA-MB-231 cells accompanied by decrease in the expression of CXCR4, MMP-2 and MMP-9 via AhR [114]. Icaritin, a prenylated flavonol glycoside, inhibits growth of MCF-7 cells in vitro and tumor growth in a xenograft model and downregulates ER expression in an AhR-dependent manner [115]. We and others have shown that the phytonutrient indole DIM represses progression of breast cancer cells in vitro and in vivo [58,116]. It inhibits AKT activation and phosphorylation of hepatocyte growth factor (HGF) and c-Met at the tyrosine residues [116] and reduces SOX4 through inducing miR-212/132-SOX4 axis [58]. Importantly, AhR ligands such as flavonoids and phytochemicals alter other pathways such as p65-NF-κB [24] and IL-6/p-signal transducer and activator of transcription 3 (STAT3) [118]. These findings and those obtained by ablation of AhR may support the conclusion that the anti-cancer activities of such natural AhR ligands are not solely attributed to the modulation of AhR signaling, raising a concern about their selectivity and specificity.

Interestingly, the blockade of AhR signaling by antagonistic compounds exerts inhibitory effects on the progression of breast cancer cells (Table 1). For instance, the quinazoline derivative (compound **12**) induces apoptosis and cell cycle arrest at the G2/M phase in MCF-7 cells [31]. Mechanistically, this AhR antagonist makes the cell cycle arrest by reducing the levels of CDC2 and CCDC25c proteins and increasing that of cyclin B1 [31]. In a comprehensive study using MCF-7 cells, Liu and team have shown that inhibition of AhR signaling by galangin upregulates the expression of BCL2-associated X protein (Bax) and decreases that of BCL-2, reduces cell viability and induces apoptosis [117]. They have also reported increases in the expressions of caspase-9, caspase-8, caspase-3, BH3 interacting domain death agonist (BID), and BCL2-binding protein (BAD), and decrease in the levels of p-PI3K and p-AKT [119]. Moreover, in the same study, they found that galangin reduces the levels of cyclin D3, cyclin B1, CDK1, CDK2, and CDK4 while increasing that of p21, p27 and p53 [119]. The 2-methyl-N-[2-methyl-4-[(2-methylphenyl)diazenyl]phenyl]pyrazole-3-carboxamide (CH223191) is a specific AhR antagonist, it represses the growth and migration of TNBCs including MDA-MB-231 and cHCI-10 PDX cells [111]. In lines, CH223191 and 2-((2-(5-bromofuran-2-yl)-4-oxo-4H-chromen-3-yl)oxy)acetamide (CB7993113) inhibit migration of Hs578T and SUM149 breast cancer cells [32]. Therefore, desired anticancer functions of AhR may be differentially induced in a ligand- and cell-dependent manner.

Several AhR lead ligands have been modified and optimized and are currently being tested in clinical trials. For example, the aminobenzothiazole (2-(4-amino-3-methlyphenyl)-5 fluorobenzothiazole (5F-203) and 2-(4-amino-3-methlyphenyl)benzothiazole (DF-203) are AhR ligands that have been tested for clinical development to treat breast cancer [112,120,121]. The aminoflavones 5-amino-2-(4-amino-3-fluorophenyl)-6,8-difluoro-7-methyl-4H-1-benzopyran-4-one (NSC-688228) and its prodrug conjugate are AhR ligands that have been evaluated in humans for breast cancer chemotherapy [90,122,123]. They exert their anticancer effects by suppressing α6-integrin-Src-AKT signaling pathways [123].

### 5.2. Colon Cancer

Several studies have indicated the therapeutic potential of AhR ligands including SAhRMs in colon cancer (Table 2). It has been shown that activation of AhR signaling by analogs of the synthetic lead compound (*Z*)-N-(4-(2-cyano-2-(3,4-dichlorophenyl)vinyl)phenyl)acetamide (compound **12g**) suppresses proliferation of HT29 cells [124]. In an independent study, Baker and coworkers have demonstrated that the methylpiperidine analogs of (*Z*)-N-(4-(2-cyano-2-(3,4-dichlorophenyl)vinyl)phenyl)acetamide exert promising anticancer properties in HT29 cells with specific activity [29]. Both synthetic piperidone analogs of curcumin RL66 (1-methyl-3,5-bis[(E)-4-pyridyl)methylidene]-4-piperidone) and RL118 (1-isopropyl-3,5-bis[(pyridine-3-yl)methylene]piperidin-4-one) induce apoptosis in a panel of colon cancer cell lines including DLD1, HCT116, LS513 and RKO [125]. Furthermore, Bap promotes the formation of DNA adduct in HCT116 cells through upregulation of CYP1A1 expression and activity, and comparable effects of Bap were observed in FHC and HT29 cells [126].

Chrysin, a natural AhR agonist, promotes apoptosis by upregulation of the proapoptotic cytokines tumor necrosis factor (TNF)-α and -β in HCT116, DLD-1, and SW837 cell lines via AhR [127]. They have also found that chrysin enhances serum-responsive elements (SRE)-driven immediate early genes (IEGs) which may contribute to the chrysin-induced apoptosis [127]. Furthermore, activation of AhR by I3C induces a dose-dependent decrease in cell viability and increases apoptosis in DLD1, HCT116, HT29, LS513, and RKO cells, and knockdown of AhR generates resistance to the chemotherapeutic actions of I3C [128]. Finally, both AhR agonists TCDD and DIM suppress tumorigeneses in the colon in the murine model of colitis-associated colon cancer [27].

Inhibition of AhR signaling by CH223191 abolishes cell cycle and proliferation arrest induced by the endogenous AhR agonist 6-formylindolo[3,2-b]carbazole (FICZ) in LoVo cells [131]. In addition, CH223191 abolishes the effects of FICZ on the expression of CDK inhibitor p27 and cyclin D1 and phosphorylation of retinoblastoma protein (Rb) [131]. Opposing results show that inhibition of AhR by CH223191 potentiates Src-mediated crosstalk between AhR and EGFR to induce ERK1/2 activation, which promotes proliferation of H508 and SNU-C4 cells [84]. In fact, there is a limited number of studies introducing mechanistic explanation for the effects of AhR ligands in colon cancer, which warrants further detailed studies.

### 5.3. Lung Cancer

Although the lung is the second organ expressing high levels of AhR after the placenta [40], a relatively limited number of screening and mechanistic studies using AhR ligands have been conducted. A recent study has indicated that the AhR agonist 11-Cl-BBQ (11-chloro-7H-benzimidazo[2,1-a]benzo[de]iso-quinolin-7-one) prompts anti-proliferative effects in H460 lung cancer cells [129]. This SAhRM makes cell cycle arrest at the G1 phase, activates p53 signaling, represses DNA replication-related pathways, and stimulates the expression of p27^Kip1^ and other cyclin-dependent kinase inhibitors [129]. In contrast, the tumor-promoting properties of certain AhR agonists have been reported. The Bap upregulates the expression of OPN in H1355 cells through AhR-NF-κB pathways interplay, which in turn augments lung tumorigenesis, and inhibition of AhR activation by DMF abolishes these effects [80]. Omeprazole preferentially regulates the expression of proteins implicated in the progression of H1975, A549, and H1299 cells [130]. It suppresses MMP-24 and upregulates activating transcription factor 4 (ATF4) and asparagine synthetase (ASNS) [130], an enzyme controls the motility of lung cancer cells by endowing stability to the β-catenin complex and modifying mitochondrial response [132]. A recent study has reported different responses in A549 cells with continuous exposure to the non-genotoxic TCDD and the genotoxic Bap [133]. They have shown that TCDD increases cell proliferation, suppresses E-cadherin, and activates epithelial-to-mesenchymal transition (EMT)-related genes, but does not induce the EMT-like phenotype, while Bap decreases cell proliferation and enhances cell migration and invasion, alters cell morphology, and induces EMT-like phenotype [133]. It has been also shown that BaP-dependent activation of AhR activates MAPK signaling leading to the induction of cell proliferation, differentiation, and apoptosis [34].

### 5.4. Other Cancers

In prostate cancer, the therapeutic potential of AhR ligands has been studied primarily in the context of androgen receptor (AR) suppression and the anti-androgenic effects. For example, the AhR indole agonists including 3-methylindole (3MI), 4MI, 2,3,7-trimethylindole (2,3,7TMI), and 7-methoxy-4-methylindole (7MeO4MI) reduce the viability of 22Rv1 cells and decrease androgen receptor levels [134]. Furthermore, flutamide and FICZ reduce the viability of LNCaP cells by acting as anti-androgen and the consequent lower levels of prostate-specific antigen (PSA), kallikrein-related peptidase 2 (KLK2), transmembrane serine protease 2, (TMPRSS2) and AR and presence of CH223191 mitigates these effects [135]. Icaritin induces AhR activation with consequent degradation of AR in LNCaP, C4-2, and 22Rv1 cells, and suppresses LNCaP growth in nude mice [136]. Comparable results revealed that activation of AhR by the polyphenol carbidopa augments AR degradation and suppresses tumor growth of LNCaP cells in vivo [137].

In hepatocellular carcinoma, flutamide inhibits the proliferation of cell line panels including human HepG2 and HuH-7, and Rat 5L via AhR [113]. In their study they found that activated AhR upregulates TGF-β1, consequently leading to induction of the cycline-dependent kinase inhibitors p15INK and p27^Kip1^ and latent-TGF-β binding protein 2 (LTBP2), bone morphogenic protein-6 (BMP6), urokinase-type plasminogen activator (PLAU), and insulin-like growth factor-binding protein 3 (IGFBP3) [113]. In pancreatic cancer, omeprazole and tranilast inhibit the invasive capacity of Panc1 and MiaPaCa2 cell lines [138]. Later study has shown that omeprazole prevents the invasion of Panc1 cells by activating a non-genomic AhR pathway, which is reliant on Jun-N-terminal kinase (JNK) and mitogen-activated kinase-kinase 7 (MKK7) [139]. Their RNAseq data shows that omeprazole induces AhR-dependent suppression of proinvasion factors including activated leukocyte cell adhesion molecule (ALCAM), long chain fatty acid CoA-synthase (CSL4), stathmin 3 (STMN3), and neuropillin 2 (NRP2) [139]. Table 3 summarizes the reported effects of AhR ligands on prostate, hepatocellular and pancreatic cancers.

## 6. AhR: A Potential Target in Cancer Immunotherapy

It is well established that AhR plays central roles in both innate and adaptive immune responses with vigorous modulatory effects, which makes AhR an attractive target for the development of cancer immunotherapies. An increasing body of data has indicated the important role of AhR in the immunosuppressive functions of IDO1 and TDO2. It has been shown that activation of AhR by kynurenine, a tryptophan metabolite derived by the IDO1/TDO2 pathway, fosters differentiation and functions of tolerogenic dendritic cells (DCs) and regulatory T cells (Treg), and consequently IL-10 secretion [142]. The immunosuppressive role of kynurenine creates a cancer-promoting microenvironment and supports cancer immune escape [142]. Furthermore, kynurenine controls the activation of tumor-associated macrophages (TAMs) through inhibition of NF-κB activation, increasing KLF4 expression and promoting CD39 expression [140]. These kynurenine-mediated effects in TMAs lead to the deterioration of CD8^+^ T cell response to glioblastoma cells by producing adenosine in cooperation with CD73 [140]. In addition, an interplay between the kynurenine pathway and NF-κB that promotes anoikis resistance in TNBCs has been identified [143]. Notably, pharmacological or genetic inhibition of AhR or TDO2 reduces resistance to anoikis and inhibits the progression of TNBCs in vitro and in vivo [133].

Moreover, inhibition of the AhR agonist kynurenine and IDO1/TDO2, and modulation of AhR signaling by antagonists has emerged as important immunotherapeutic perspective. It has been shown that depletion of kynurenine by administering engineered kynureninase (KYNase) exerts substantial therapeutic effects in animal models when combined with certain checkpoint inhibitors or vaccines for the treatment of different cancers including B16-F10 melanoma, 4T1 breast carcinoma, and CT26 colon carcinoma [144,145]. The KYNase increases effector T cells and accumulation of CD8^+^ T cells in the tumor and increases the levels of interferon-γ (IFN-γ) in the TME [144,145]. Currently, selective IDO1/TDO2 inhibitors are being evaluated at different clinical phases to treat various cancers such as epacadostat, navoximod, BMS-986205, and PF-06840003 [35]. These IDO1 inhibitors work primarily by enhancing/restoring the immune response [35,146]. As discussed in previous sections, inhibition of AhR functions by antagonists as a therapeutic potential has been investigated in different cancers and several mechanisms have been proposed. However, a limited number of studies, if any, have investigated the effects of such modulation on the IDO1/TDO2-kynurenine-AhR pathway, indicating that the research in this specific field is still in its infancy and warrants further investigation.

The AhR enhances the release of amphiregulin (AREG) and specific chemokines including granulocyte-colony stimulating factor (G-CSF), CXCL1/2/5, and CCL2/5 in the TME in human breast cancer tissue bearing BRCA1 mutation, which may facilitate the activation of protumorigenic and angiogenic TAMs [36]. In their study, they have also found that inhibition of AhR by CH223191 reduces the secretion of AREG in MDA-MB-468 and HCC1937 cells and phosphorylation of EGFR in HCC1937 cells. Interestingly, CH223191 exerts synergistic anti-cancer effects with erlotinib, an EGFR inhibitor, in BT20, MDA-MB-468, and HCC1937 cells [36]. These results may introduce targeting the AhR–AERG axis as a potential therapeutic strategy for BRCA1-associated breast cancer, and potentially other cancers expressing high levels of EGFR. In the context of the expression of chemokines and chemokine receptors, Takenaka and coworkers have shown that kynurenine stimulates AhR in TAMs and promotes CCR2 expression, and consequently drives recruitment of TAMs in response to CCL2 [140]. The AhR agonist luteolin decreases the expression of the prometastatic markers CXCR4, MMP-2, and MMP-9, which was abolished by chemical inhibition of AhR by stemregenin 1 (SR1) [114]. In addition, omeprazole decreases the expression of CXC4 in TNBCs via AhR and mitigates their metastasis to the lung in a mouse model [92]. Furthermore, activation of AhR in vitro by TCDD induces IL-8 expression in TNBCs and ER-positive breast cancer cells in an AhR- and RelB-dependent manner [147]. Both DMBA and FICZ elevate the expression of CYP1A1, p19, CCL20, and IL-36γ mRNA in normal human keratinocytes in vitro [148]. They have also found that conditional deletion of AhR in mice reverses these effects and reduces IL-17, which collectively contributes to the reduced number of squamous cell carcinoma lesions [148].

A recent study has shown that IL-2, through activation of STAT5-5-hydroxytryptophan (5-HTP)-AhR pathway, induces CD8^+^ T cell exhaustion in the TME [149]. They have also shown that AhR translocation activated by 5-HTP induces tumor-specific CD8^+^ T cell exhaustion accompanied by upregulation of programmed cell death protein 1 (PD-1), lymphocyte activating 3 (LAG3), and CD39, and downregulation of cytokines, and consequently causing T cells dysfunction in the TME [149]. Furthermore, recent data indicates a correlation between AhR expression and immune inhibitors including colony-stimulating factor 1 receptor (CSF1R) and galectin 9 (LGALS9) in uterine carcinosarcoma and IL10RB in testicular germ cell tumors [150]. Remarkably, they have also found a positive correlation between AhR expression and immune stimulators including TMEM173 and TNF superfamily member 13 (TNFSF13) in testicular germ cell tumors as well as CD48 and TNFRSF25 in uveal melanoma [150]. Further correlation between AhR and tumor mutational burden (TMB) and microsatellite instability (MSI) has been reported in both colon adenocarcinoma and thymoma, and a correlation between AhR and MSI in colon and rectum adenocarcinomas has been reported [150]. Lewis lung cancer-inoculated mice treated with ANF showed a reduction in PD ligand 1 (PD-L1) expression and suppressed tumor growth coupled with elevated levels of the cytotoxic cytokine IFN-γ and CD8^+^ T cell numbers in the lungs [37,151]. The AhR agonist BaP increased PD-L1 (B7-H1) expression in non-small cell lung cancer cells; this impact was AhR-dependent and was reduced by the AhR antagonists CH223191 and ANF [151].

## 7. Conclusions

A growing body of data suggests AhR as a promising molecular target for the development of new anticancer agents owing to its vigorous modulatory effects on several physiological processes. It recognizes structurally diverse exogenous ligands that exhibit their effects on cancer by inducing or inhibiting the canonical, non-canonical, and/or non-genomic AhR pathways. Some of these ligands are being examined for clinical development as potential anticancer drugs with favorable outcomes. Yet, concerns related to the promiscuity of AhR recognition to ligands and the varying effects of the agonistic and antagonistic compounds on cancer. From the ligand point of view, these differences may be attributed to different modes of interaction between the ligands and the residues of the AhR binding site. These modes of interaction, most likely, induce changes in AhR (physical, chemical, etc.), that determine the partner in the heterodimer complex, and consequently, the AhR pathway(s) and interactions with other signaling pathways. These events collectively induce selective gene expression and regulatory mechanisms that determine the outcome of AhR activation to promote or suppress cancer in certain tissues. Therefore, AhR ligands are worth additional structural, pharmacological, pharmacokinetic, and mechanistic investigations to disclose the factors responsible for these differences. In contrast to the promiscuity of AhR, the selectivity of certain ligands has been demonstrated in different cancer cell lines. It is believed that such selectivity is advantageous and may lead to developing specific therapies; however, studies to unravel the underlying mechanisms are a prerequisite.

A considerable deal of discrepancies in the anticancer effects of similar classes of AhR ligands in similar cancer cell lines has been reported. Such discrepancies may be attributed, at least partially, to the experimental conditions. For instance, the exposure time, dose, and concentration of AhR ligands may cause significant differences even in the same cell line. Furthermore, variation in the metabolism rate of the ligands within the cells can create different outcomes, especially when the ligand is long-acting and accumulates within the cell or the tissue. It is our opinion that optimizing the structure–activity axis of the ligands to ensure potent activation by agonists or inhibition by antagonists while ensuring fast metabolic degradation is advantageous.

The recent paradigm has clearly indicated the immunosuppressive function of IDO1/TDO2 through forming the endogenous AhR agonist kynurenine. The kynurenine-activated AhR leads to immunosuppression and tumor-promoting microenvironment. Furthermore, the IDO1/TDO2-AhR signaling pathway endows cancer cells with the capacity for evading immune surveillance and escaping immune responses. Therefore, pharmacological targeting of the IDO1/TDO2-AhR pathway may offer a promising immunotherapeutic strategy. Although still far-reaching, specific modulation of AhR pathways in TAM and cancer-specific CD8^+^ T cells by ligands may be considered for future investigation. Finally, combined therapies using AhR ligands with lower doses of conventional cancer drugs and chemotherapies may offer a favorable strategy for control of the progression and treatment of cancer.

## Figures and Tables

**Table 2:** Exogenous AhR ligands in colon and lung cancer.

Compound	Ligand	Response	Cells	Refs.
**Colon Cancer**				
Compound **12g ** (acetamide)	Agonist	Proliferation	HT29	[124]
RL66	Agonist	Apoptosis, Proliferation	DLD1, HCT116, LS513, RKO	[125]
RL118	Agonist	Apoptosis, Proliferation	DLD1, HCT116, LS513, RKO	[125]
Bap	Agonist	DNA damage	HCT116, FHC, HT29	[126]
Chrysin	Agonist	Apoptosis, Proliferation	HCT116, DLD-1, SW837	[127]
I3C	Agonist	Apoptosis, Proliferation	DLD1, HCT116, HT-29, LS513, and RKO	[128]
**Lung Cancer**				
11-Cl-BBQ	Agonist	Cell Cycle Arrest, Proliferation	H460	[129]
Bap	Agonist	Proliferation, Migration, Invasion, OPN	H1355, A549	[80,113]
Omeprazole	Agonist	ASNS, ATF4	H1975, A549, H1299	[130]

**Table 3:** Exogenous AhR ligands in various cancers.

Compound	Ligand	Response	Cells	Refs.
**Prostate**				
3MI	Agonist	Proliferation	22Rv1	[134]
4MI	Agonist	Proliferation	22Rv1	[134]
2,3,7TMI	Agonist	Proliferation	22Rv1	[134]
7MeO4MI	Agonist	Proliferation	22Rv1	[134]
Flutamide	Agonist	Proliferation	LNCaP, PC3	[113,135]
Carbidopa	Agonist	Proliferation, Migration, Growth	LNCaP	[137]
Icaritin	Agonist	Apoptosis, Proliferation, Growth	LNCaP, C4-2, 22Rv1	[136]
**Hepatocellular Carcinoma**				
FDI-6	Agonist	Tumorsphere formation	HepG2	[110,140]
Flutamide	Agonist	Proliferation	HepG2, HuH-7, 5L	[113]
**Pancreatic Cancer**				
Omeprazole	Agonist	Invasion	Panc1, MiaPaCa2	[138]
Tranilast	Agonist	Invasion	Panc1, MiaPaCa2	[138]
Carbidopa	Agonist	Growth	PDAC	[141]
**Ovarian**				
Compound **12a** (acrylonitrile)		Proliferation	A2780	[29]
